# Physically Crosslinked Poly(methacrylic acid)/Gelatin Hydrogels with Excellent Fatigue Resistance and Shape Memory Properties

**DOI:** 10.3390/gels10070444

**Published:** 2024-07-04

**Authors:** Vukasin Ugrinovic, Maja Markovic, Bojan Bozic, Vesna Panic, Djordje Veljovic

**Affiliations:** 1 Innovation Center of Faculty of Technology and Metallurgy, University of Belgrade, 11000 Belgrade, Serbia; mmarkovic@tmf.bg.ac.rs (M.M.); vpanic@tmf.bg.ac.rs (V.P.); 2 Institute of Physiology and Biochemistry “Ivan Đaja”, Faculty of Biology, University of Belgrade, 11000 Belgrade, Serbia; 3 Faculty of Technology and Metallurgy, University of Belgrade, 11000 Belgrade, Serbia

**Keywords:** hydrophobic interactions, mechanical properties, shape memory, self-healing

## Abstract

Hydrogels endure various dynamic stresses, demanding robust mechanical properties. Despite significant advancements, matching hydrogels’ strength to biological tissues and plastics is often challenging without applying potentially harmful crosslinkers. Using hydrogen bonds as sacrificial bonds offers a promising strategy to produce tough, versatile hydrogels for biomedical and industrial applications. Poly(methacrylic acid) (PMA)/gelatin hydrogels were synthesized by thermally induced free-radical polymerization and crosslinked only by physical bonds, without adding any chemical crosslinker. The addition of gelatin increased the formation of hydrophobic domains in the structure of the hydrogels, which acted as permanent crosslinking points. The increase in PMA and gelatin contents generally led to a lower equilibrium water content (WC), higher thermal stability and better mechanical properties. The values of tensile strength and toughness reached up to 1.44 ± 0.17 MPa and 4.91 ± 0.51 MJ m^−3^, respectively, while the compressive modulus and strength reached up to 0.75 ± 0.06 MPa and 24.81 ± 5.85 MPa, respectively, with the WC being higher than 50 wt.%. The obtained values for compressive mechanical properties are comparable with super-strong hydrogels reported in the literature. In addition, hydrogels exhibited excellent fatigue resistance and biocompatibility, as well as great shape memory properties, which make them prominent candidates for a wide range of biomedical applications.

## 1. Introduction

Hydrogels are three-dimensional hydrophilic networks of polymers capable of retaining large amounts of water or biological fluids. Due to their highly hydrated, porous structure resembling living tissues, hydrogels have been mainly investigated for biomedical and pharmaceutical applications. However, over the past few years, the application potential of hydrogels has rapidly expanded and now includes sensors and actuators [1,2], soft machines [3], and flexible electronics [4]. In all these applications, hydrogels are commonly subjected to different types of dynamic stresses such as stretching, compression, and torsion, which sets strict requirements regarding the mechanical properties of the hydrogels [5]. Although scientists’ efforts to create mechanically competent hydrogels have resulted in a substantial improvement in physicochemical properties, the developed hydrogels are still largely inferior to biological tissues and solid-engineered plastics in many aspects, while many of them require time-consuming multi-step synthesis procedures often involving toxic compounds for crosslinking the structure of the hydrogel [6,7]. For example, the tensile strength of biological tissues such as tendons and ligaments reaches up to 49 and 65 MPa, respectively, while the tensile strength of engineering plastics is within the range from ten to several tens of megapascals [6]. On the other hand, the tensile strength of hydrogels is relatively low, often lower than 1 MPa for hydrogels with a water content higher than 50%.

Nevertheless, remarkable progress in the mechanical properties of hydrogels has been achieved over the last few decades by developing interpenetrating network hydrogels (IPNs), which are composed of two or more independently crosslinked networks interlaced at a molecular level [8]. The constituting networks of IPNs are typically crosslinked by chemical bonds, which can enhance the mechanical strength of hydrogels but result in poor deformation-tolerant and fatigue-resistant performance, as the breakage of covalent crosslinks leads to irreversible structural damage [9]. On the other hand, the synthesis of fully physical networks, crosslinked by multiple dynamic bonds, has been recognized as an effective approach to obtain hydrogels with high strength, toughness, fast shape recovery, and fatigue resistance [10]. The implementation of hydrogen bonds as sacrificial bonds is an effective strategy to construct tough, promising hydrogels for biomedical and industrial applications due to the widely distributed bond strength and insensitivity to the neutral ionic environment of hydrogen bonds [11]. However, hydrogels with only hydrogen bonds as an energy-dissipating mechanism are often characterized by limited mechanical properties, as water molecules compete to form hydrogen bonds in aqueous conditions [12]. In recent years, several hydrogels with high toughness have been developed by applying relatively stable hydrogen-bonding pairs and hydrophobic side groups to protect hydrogen bonds [11,13,14]. The structure of such hydrogels consists of dense polymer domains which are connected by hydrogen bonds and stabilized by hydrophobic interactions, hindering water penetration and ensuring stability in aqueous environments. These domains act as permanent crosslinks which impart dimensional stability and outstanding mechanical properties to the hydrogels.

Gelatin is a natural polymer that has been widely applied in biomedicine due to its low price, excellent biocompatibility, biodegradability, and ability to promote cell adhesion, proliferation, and differentiation [15]. However, gelatin-based hydrogels suffer from low mechanical properties and rapid dissolution in an aqueous environment at physiological temperatures, which often requires creative approaches to obtain functional gelatin-based hydrogels.

Poly(methacrylic acid) (PMA) is a nontoxic polymer which can be easily optimized to obtain hydrogels with different porosity, swelling ability, and mechanical properties [16]. PMA is also highly hydrophilic polymer with many carboxylic groups in the structure, which can act as hydrogen bond donors and acceptors, and also contain α-methyl groups which can interact with hydrophobic groups and significantly improve the mechanical properties of PMA-based hydrogels [17].

Recently, there has been a growing trend of combining methacrylic acid (MA) with different vinyl monomers to take advantage of the hydrophobicity of the α-methyl group on MA and create hydrogels with improved mechanical properties. Hu and coworkers combined MA with N, N-dimethylacrylamide (DMAA) to obtain poly(MA-co-DMAA) hydrogels with outstanding mechanical properties [12]. The structure of the hydrogels was composed of polymer-rich clusters as the main crosslinking points, which contained macromolecular chains bonded by hydrogen bonds and stabilized by hydrophobic interactions, embedded in a loose covalent network. The excellent mechanical performance of the hydrogels was attributed to an optimum balance between the density of permanent crosslinking and the strength of hydrogen-bonded clusters stabilized by hydrophobic interactions. However, this was possible only in acidic conditions (pH = 3). In pure water, the hydrogels became highly swollen with a significant deterioration in mechanical properties. Also, Zhang and coworkers prepared mechanically strong hydrogels by the copolymerization of 1-vinylimidazole (VI) and MA [14]. The mechanical properties of the poly(VI-co-MA) hydrogels were multiple times higher than the corresponding hydrogels obtained by the copolymerization of VI and acrylic acid (AA), which was attributed to the stabilizing effect of hydrophobic interactions on hydrogen bonds. Although the hydrogels demonstrated great mechanical properties, the fabrication process was very long (1 week) and involved the use of organic solvents, which could negatively affect the biocompatibility of the hydrogels. Similarly, Zhou et al. prepared exceptionally strong hydrogels through the photo-initiated copolymerization of MA and N-(pyridin-2-yl)acrylamide. However, the synthesis process also required the use of an organic solvent [18]. In another example of the successful combination of MA with vinyl monomers, Wang et al. synthesized poly(methacrylamide-co-MA) hydrogels, which contained 50–70% water in the structure and exhibited ultra-high stiffness and excellent tensile strength [13]. The high stiffness was attributed to the dense crosslinking caused by the compact hydrogen bonds between the carboxylic acid and amide groups, which were stabilized by the hydrophobic methyl motifs.

In a previous study, the authors reported PMA/gelatin IPN hydrogels, utilizing N,N-methylenebisacrylamide (MBA) as the crosslinker, yielding a biocompatible, biodegradable, and porous structure [19]. Gelatin was chosen as a highly biocompatible element to enhance the bioactivity and biocompatibility of the hydrogels. On the other hand, PMA was used as a network-forming component, which formed the framework of the hydrogel and allowed the simple manipulation of network parameters. The synthesis of the hydrogels was performed via thermally induced free-radical polymerization by using sodium hydroxide as an MA-neutralizing agent. In this way, it was possible to obtain porous hydrogels with a high water content appropriate for biomedical applications, and also to reach reasonable values of compressive mechanical strength (up to 16 MPa). However, the use of sodium hydroxide partially disrupted the formation of hydrophobically stabilized hydrogen bonds and reduced the mechanical and shape memory properties of the hydrogels. In addition, the use of MBA as a crosslinker might increase cytotoxicity, while increased covalent crosslinking reduces the recoverability and self-healing properties of these hydrogels. Similarly, Zhang et al. reported PMA/gelatin hydrogels using a low-temperature UV polymerization procedure, followed by drying at room temperature and swelling for 12 h [20]. During this process, gelatin initially forms triple-helix junctions in the first step due to the pre-polymerization temperature being below the UCST of the gelatin/MA mixture, followed by UV irradiation inducing the polymerization of MA. The influence of the pre-polymerization temperature on the mechanical properties of the hydrogels was significant, with the tensile strength increasing from 0.5 to 4.5 MPa as the temperature decreased from 12 to 2 °C. This increase was attributed by the authors to the higher crosslinking density of gelatin at lower temperatures, leading to the formation of a more densely crosslinked PMA network during polymerization. The hydrogels that were presented therein exhibited excellent tensile mechanical properties, self-healing capability, and favorable biocompatibility. Nevertheless, the described preparation process required three sequential steps to achieve mechanically robust hydrogels. So, the procedure could be unpractical for large-scale production. The room-drying phase, which could extend over several days, coupled with an additional 12 h of swelling, significantly lengthens the fabrication process.

In this study, we employed a thermally induced free-radical polymerization to create PMA/gelatin (PMAG) hydrogels, crosslinked exclusively through physical bonds, using a straightforward, one-pot procedure. Specifically, the synthesis temperature being higher than 40 °C prevents the formation of gelatin triple-helix junctions (as it is above the UCST of the gelatin/MA mixture), resulting instead in the formation of a homogeneous network of PMA and gelatin. Through the applied approach, it was possible to obtain high-strength hydrogels containing more than 50 wt.% water in the structure and with shape memory properties.

## 2. Results and Discussion

### 2.1. The Design of IPN Hydrogels

**Figure 1:**
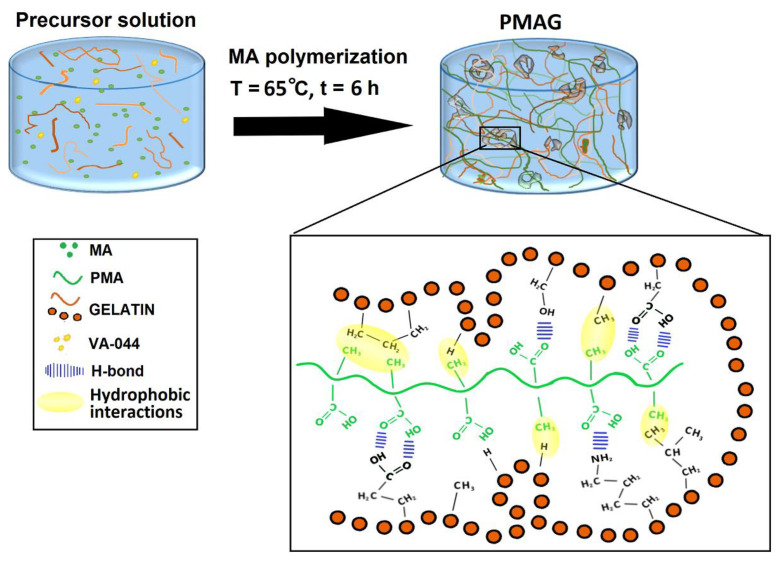
Synthesis process and possible interactions in PMAG hydrogels.

A schematic illustration of the preparation process of PMAG hydrogels is presented in Figure 1. After the thermally induced initiation of the free-radical polymerization reaction, the PMA chains arose from MA monomers in the presence of gelatin molecules previously dissolved in the precursor solution. The polymerization of MA led to multiple intermolecular hydrogen bonds between PMA and gelatin, which caused the formation of polymer-rich aggregates stabilized by the hydrophobic interactions between the two polymers [12]. The proposed mechanism of interaction is similar in many aspects to that of lipids and detergents. The hydrophobic groups in PMA and gelatin may pack closely together to form the core of the structure, and the hydrophilic groups point toward the polar phase. Gelatin is composed of many hydrophobic amino acids, such as glycine, alanine, proline, leucine, etc., accounting for more than 60% of the total amino acid content, which can react with α-methyl groups in PMA and stabilize hydrogen bonds formed between the PMA and gelatin [21]. In this way, it is possible to obtain polymer-rich clusters of different sizes, connected via hydrogen bonds and hydrophobic interactions, which may act as the main crosslinking points in the structure of the hydrogels. Polymer-rich clusters are surrounded by a relatively loose polymer-poor phase. A total of nine hydrogels were synthesized with different MA and gelatin concentrations, as well as a single network PMA.

### 2.2. The Degree of Conversion

**Figure 2:**
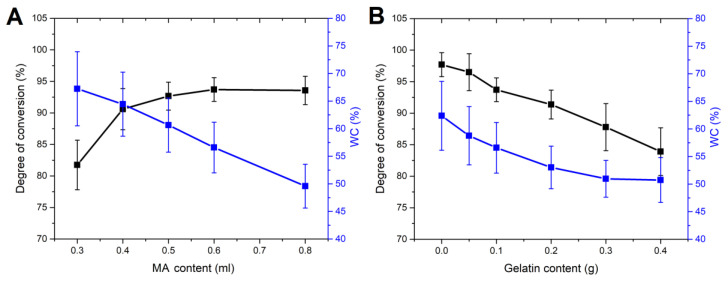
The influence of MA (**A**) and gelatin (**B**) content on DC and water content (WC).

The degree of conversion (DC) directly affects the physical and mechanical properties of hydrogels, as it shows how much monomer is incorporated into the structure of the polymer. As demonstrated in Figure 2, the increase in MA content led to a higher DC, while the increase in gelatin content oppositely affected the DC. For example, the DC of PMA30G10 was 82%, while the DC of PMA40G10 was 91%. The reason for the relatively low DC of PMA30G10 was probably the insufficient amount of initiator with regard to the total volume of the precursor solution, which led to a higher amount of monomer being unreacted and eventually lost. Since the initiator/monomer ratio was the same in all cases, a reduction in both initiator and monomer concentrations within the reaction mixture resulted in a decreased average distance between molecules. Consequently, there was a lower probability of the generated free radicals reacting with the monomer molecules.

When the monomer content exceeded 0.5 mL, there was enough initiator to effectively initiate the growing of PMA chains able to generate enough entanglements and interactions with gelatin, in this way forming a stable network with very low leaching of monomer, oligomers, and unbonded PMA. A further increase in MA and initiator concentrations did not lead to an increase in DC. Furthermore, the DC of PMA80G10 was slightly lower than the DC of PMA60G10, which could be explained by viscosity limitations. During the polymerization, as the reaction progressed, the concentration of polymer increased, which caused a diminished mobility of unreacted monomers [22]. At some point in the reaction, the concentration of polymer was high enough to drastically reduce the mobility of unreacted monomers and inhibit their incorporation into a growing chain. Similarly, the increase in gelatin content increased the viscosity of the reacting solution, thus reducing the mobility of unreacted monomers and the DC.

### 2.3. Water Content

Hydration and water content are two of the most important properties of hydrogels. The water in the structure not only improves the biological response of hydrogels but also reduces the friction and transfers the load through the structure. For example, the water content of connective tissues such as cartilage is 60–85%, that of skin is 20–70%, depending on the skin layer, and that of muscles is 75–80% [23,24,25]. On the other hand, the increase in water content softens hydrogels and reduces their mechanical strength. The WC of the PMAG hydrogels presented here was within the range of 50–70%, which is similar to the WC of the biological tissues. As seen from Figure 2A, the increase in MA content led to an increase in WC up to an MA content of 0.6 mL, while afterwards, it decreased negligibly. This result could be connected to the fact that at lower MA content, the network was not efficiently formed, due to the absence of enough entanglements. As the monomer concentration increased, the amount of polymer formed per unit of volume also increased, as well as the possibility for effective crosslinking via interactions with other PMA chains and gelatin and via chain entanglement. Additionally, it has been demonstrated that the rate of chain transfer increases with rising monomer concentration, along with an increase in branching and self-crosslinking reactions. Consequently, this augments the crosslinking degree, reducing the WC with an increase in MA content [16]. On the other hand, the increase in gelatin concentration decreased the WC due to the heightened presence of hydrophobic domains, thereby intensifying the crosslinking degree and impeding water penetration into the structure.

### 2.4. Microstructure

**Figure 3:**
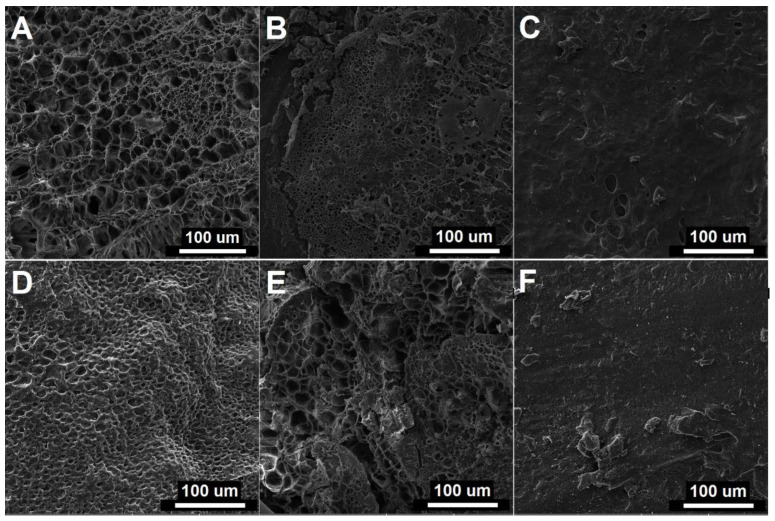
Cross-section micrographs of freeze-dried hydrogels: PMA60G0 (**A**), PMA60G20 (**B**), PMA60G40 (**C**), PMA30G10 (**D**), PMA60G10 (**E**), and PMA80G10 (**F**).

The microstructural characteristics of the hydrogels, as investigated by SEM, were consistent with the WC results. Increasing gelatin content significantly reduced the porosity of the hydrogels, as illustrated in Figure 3A–C. For instance, the microstructure of PMA60G10 exhibited a porous architecture with pores ranging from 10 to 50 µm, whereas the PMA60G40 sample displayed a comparatively compact structure. Similar trends were observed with increasing PMA content, where the sample with the lowest PMA content showed relatively high porosity (Figure 3D), while higher PMA content led to decreased porosity and increased compactness (Figure 3E,F). The formation of pores in the hydrogel structure occurred during the freezing process, wherein entrapped water solidified into ice crystals subsequently sublimated during freeze drying. Hence, the microstructure of the hydrogels was significantly influenced by their water content.

### 2.5. Physicochemical Structure of Hydrogels

**Figure 4:**
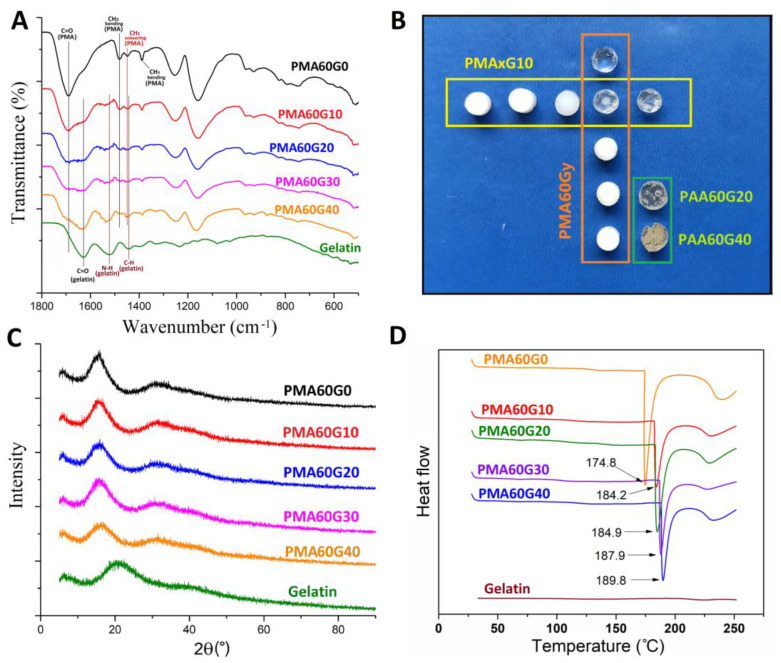
FTIR spectra of neat PMA hydrogel, PMAG hydrogels, and gelatin (**A**). Photographs of the obtained IPN hydrogels (yellow rectangle: x = 30, 40, 50, 60, and 80, from left to right; orange rectangle: y = 0, 10, 20, 30, and 40, from top to bottom) (**B**). XRD spectra of neat PMA hydrogel, PMAG hydrogels, and gelatin (**C**). DSC curves of neat PMA hydrogel, PMAG hydrogels, and gelatin (**D**).

Figure 4A presents the FTIR spectra of the single-network PMA hydrogel, gelatin, and the synthesized hydrogels. The spectrum of pure PMA in the fingerprint region showed the following: a strong peak at 1690 cm^−1^ assigned to the C=O stretching vibration of carboxylic groups; small peaks at 1481 cm^−1^ (CH3 asymmetric bending), 1449 cm^−1^ (CH2 scissoring), and 1388 cm^−1^ (CH3 symmetric bending); a peak at 1252 cm^−1^ (C-C-O stretching); and a peak at 1158 cm^−1^ attributed to C-O stretching [26].

The FTIR spectrum of the gelatin fingerprint region is characterized by strong peaks at 1628, 1522, and 1234 cm^−1^ attributed to νC=O and νCN stretching vibrations of the polypeptide backbone, δNH and νCN vibrations of amide II, and νCN and δNH vibrations of the amide III band, respectively [27].

Compared to the FTIR spectra of the pure PMA and gelatin, the PMAG hydrogels exhibit characteristic spectral bands from both components according to their content in the hydrogels. With the increasing gelatin content, the peak assigned to C=O stretching vibrations of the polypeptide backbone, coupled with the peak assigned to the C=O stretching of PMA carboxyl groups, presents a significant decrease in intensity of both peaks and forms a single broader peak between 1590 and 1750 cm^−1^. In addition, the peak corresponding to N-H bending in the gelatin backbone shifts from 1522 to 1540 cm^−1^. The observed spectral shifts indicate interactions between the carboxyl groups of PMA and amide groups of gelatin. The small blue shift (from 1628 to 1633 cm^−1^) of the C=O stretching vibrations of amide groups in gelatin could be attributed to a charge redistribution and a decrease in the p–π conjugation effect of the amide group after the formation of the hydrogen bond in PMAG [17]. The observed shifts unambiguously indicate the formation of hydrogen bonds [28]. In addition, the PMAG hydrogels also exhibit small shifts related to CH3 vibrations of PMA, which increase as the content of gelatin increases (1481 → 1477 → 1475 → 1473 → 1471 cm^−1^). Meanwhile the peak for CH2 scissoring (1449 cm^−1^) of PMA coupled with the sp3 C-H vibrations peak of gelatin (1442 cm^−1^), forming a joint peak. This suggests potential hydrophobic interactions between PMA and gelatin.

A decrease in the transparency of the hydrogels with the increase in the gelatin/PMA ratio was another indication of the formation of hydrophobic interactions (Figure 4B). Hydrogels with lower gelatin-to-PMA ratios (PMA60G0, PMA60G10, PMA80G10) were transparent, while the others were opaque. Interestingly, hydrogels synthesized with poly(acrylic acid) (PAA) instead of PMA (PAA60G20 and PAA60G40) were also transparent, confirming that α-methyl groups on PMA and hydrophobic interactions affected the transparency of the hydrogels. In addition, the PAA60G20 and PAA60G40 hydrogels had a soft, gum-like structure, indicating a high mobility of the chains and a low degree of crosslinking. On the other hand, PMAG hydrogels formed a strong, three-dimensional structure, emphasizing the role of the α-methyl group in reducing chain mobility and creating hydrophobic domains which acted as crosslinking points. Zhang et al. observed the same decrease in transparency in gelatin/poly(MA-co-AA) with the increase in MA content as a consequence of microscale phase separations [11]. In addition, the increase in MA content led to a gradual transition of the IPN structure from a homogeneous network to a heterogeneous structure with phase separations of different domain sizes. A similar behavior was observed in our previous work on chemically crosslinked PMAG hydrogels, which indicated the formation of stable hydrophobic domains in the structure of the PMAG hydrogels [19]. On the other hand, due to an absence of hydrophobic groups on AA and a high number of hydrophilic groups, the AA and gelatin were relatively homogeneously mixed and formed a uniform structure which was transparent in visible light.

The XRD patterns of the PMA, gelatin, and PMAG hydrogels are shown in Figure 4C. Pure gelatin exhibited an amorphous structure with a very low degree of crystallinity, with a broad diffraction peak centered around 20°, which could be attributed to reconstructed triple-helix junctions [29]. Similarly, the PMA hydrogel revealed an amorphous structure with so-called short range order, since a prominent peak at 15° and a blunt peak at 31° were observed [30]. For the PMAG hydrogels, the typical peak of gelatin disappeared, while the strong peak of PMA decreased in intensity. This implies that the molecules of gelatin were rearranged and uniformly distributed in the PMA network while decreasing its crystallinity. Contrary to previous assumptions, gelatin molecules did not form triple-helix junctions in the PMAG network [19,31] but were completely embedded in the PMA structure due to the high degree of physical interactions with the molecules of PMA.

The DSC thermograms of the synthesized hydrogels, demonstrated in Figure 4D, exhibit two endothermic peaks, a sharp one in the range from 174 to 190 °C and a wide one around 240 °C. Both peaks are ascribed to different degradation phases of the hydrogel networks. The first peak is attributed to the dehydration of carboxyl groups and formation of cyclic polyanhydride [32], while the second one is related to the further degradation of the main PMA chains, including decarboxylation, depolymerization, etc. [32,33]. The increase in gelatin content shifted the first degradation stage towards higher temperatures, indicating an increase in the thermal stability of the PMAG hydrogels. The increase in the thermal stability of the hydrogels is another piece of evidence of the successful and efficient incorporation of the gelatin into the structure of the PMA, which enabled a higher degree of crosslinking due to hydrogen bonds and hydrophobic interactions.

### 2.6. Mechanical Properties

**Figure 5:**
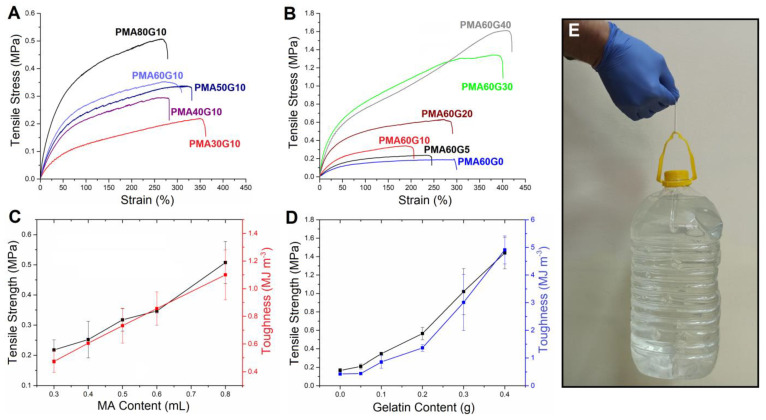
Tensile stress–strain curves of PMAG hydrogels versus MA (**A**) and gelatin (**B**) content. The dependence of tensile strength and toughness of PMAG hydrogels on MA (**C**) and gelatin (**D**) content. A demonstration of excellent mechanical properties of the PMA60G40 by carrying 5 kg bottle filled with water (**E**).

To study the mechanical properties of the hydrogels, static tensile tests and unconfined static and dynamic compression tests were performed. Tensile stress–strain curves as well as the trends in tensile strength (*σ_t_*) and toughness (*U_t_*) versus MA and gelatin contents are presented in Figure 5. The tensile strength and toughness significantly increased as the MA concentration increased from 0.3 to 0.8 mL/mL. For example, the values of *σ_t_* and *U_t_* of PMA80G10 were 0.51 ± 0.07 MPa and 1.10 ± 0.18 MJ m^−3^, respectively, which are ≈2.3 times greater than the corresponding values of PMA30G10. Similarly, the increasing gelatin concentration led to an improvement in *σ_t_* and *U_t_*, reaching values of 1.44 ± 0.17 MPa and 4.91 ± 0.51 MJ m^−3^, respectively, at a gelatin concentration of 0.4 g/mL. These values represent a significant leap in mechanical properties, as the pure-PMA hydrogel exhibited values of 0.17 ± 0.02 MPa and 0.42 ± 0.03 MJ m^−3^ for *σ_t_* and *U_t_*, respectively. The cylindrical PMA60G40 sample with a diameter of 5 mm could easily support a bottle with a weight of 5 kg, confirming its outstanding mechanical strength (Figure 5E).

The presented values for *σ_t_* and *U_t_* were relatively high and comparable with the values of different strong gelatin-based hydrogels with similar WC (Table 1). For example, Hou et al. prepared gelatin/poly(acrylamide-co-hexadecyl methacrylate) IPN hydrogels stabilized by macromolecular microspheres as crosslinking centers for hydrophobic association [34]. The hydrogels achieved a tensile strength of 1.48 MPa and toughness of 8.83 MJ m^−3^; however, no information about the WC of the hydrogels was given. Similarly, in the work of Zhang and coworkers, PMA/gelatin hydrogels achieved a tensile strength of almost 5 MPa, with the WC of the hydrogels being around 45% [20]. Also, He et al. obtained exceptionally strong gelatin hydrogels by immersing gelatin in a (NH_4_)_2_SO_4_ solution, which promoted the hydrophobic interactions between gelatin molecules [35]. The obtained hydrogels achieved compressive and tensile strengths of up to 12 and 3 MPa, respectively, at 50% WC. On the other hand, Tang and coworkers synthesized a series of gelatin/poly(N-hydroxyethyl acrylamide) IPN hydrogels with excellent mechanical and self-healing properties [36]. The tensile strength of the hydrogels varied from 0.74 to 2.33 MPa depending on the composition and WC. The WC of the hydrogel with the highest *σ_t_* was 30%. Zhang et al. prepared gelatin/poly(MA-co-AA) hydrogels with different MA/AA ratios [11]. The tensile strength of the obtained hydrogels was within the range of 0.1–7.0 MPa, while the WC was ≈ 40%. Also, Umit Gulyuz obtained Triton X-100-grafted PMA hydrogels with *σ_t_* and *U_t_* reaching up to 3.6 ± 0.2 and 20 ± 2 MJ m^−3^, respectively; however, they only contained 26 ± 3% water in the structure [37]. It is obvious that the WC had a significant influence on the mechanical properties of the hydrogels, and the obtained hydrogels presented an optimal balance between the mechanical properties and WC, while being significantly stronger than the gelatin-based hydrogels commonly reported in the literature.

**Figure 6:**
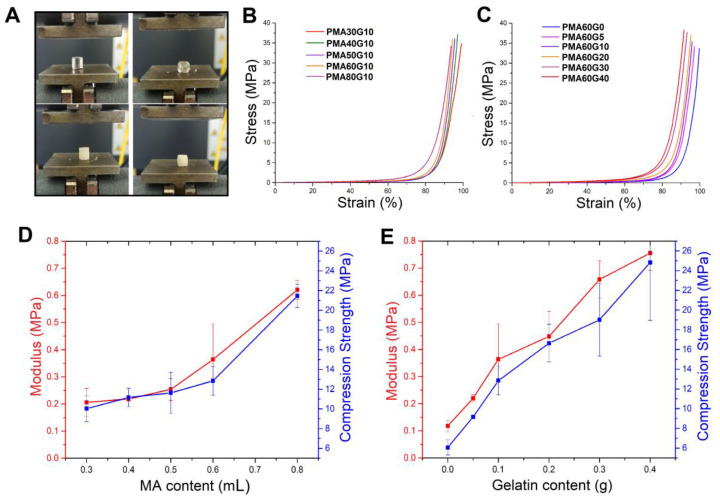
Photographs of PMA60G0 (I) and PMA60G40 (II) hydrogels before and after uniaxial compression test (**A**). Stress–strain curves for PMAG hydrogels with different MA content (gelatin content is 0.1 g/mL) (**B**) and different gelatin content (MA content is 0.6 mL/mL) (**C**). The dependence of modulus and compression strength of PMAG hydrogels on MA content (**D**) and gelatin content (**E**).

**Figure 7:**
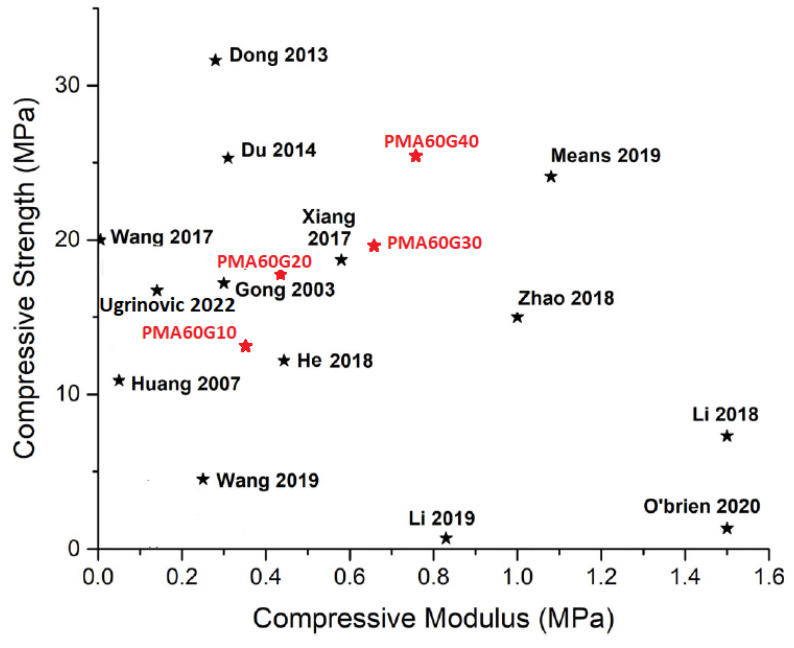
A summary of compressive mechanical properties of previously reported hydrogels (black font) with WC > 50% and comparison with the hydrogels obtained in this work (red font) [19,35,40,41,42,43,44,45,46,47,48,49,50,51].

The compressive mechanical properties followed a similar trend to the tensile strength and toughness (Figure 6). The compressive stress–strain curves had a typical exponential shape, characteristic of dynamically crosslinked hydrogels. Since the hydrogels did not break down despite almost complete compression, the values for compressive strength were taken at 90% deformation. The increase in MA and gelatin contents led to a significant increase in compressive modulus (*E_c_*) and strength (*σ_c_*). For example, the *E_c_* and *σ_c_* values of PMA80G10 were 0.62 ± 0.03 MPa and 21.45 ± 1.17 MPa, which were 3 and 2 times higher than the corresponding values of PMA30G10. Similarly, the *E_c_* and *σ_c_* of PMA60G40 were 0.75 ± 0.06 MPa and 24.81 ± 5.85 MPa, which were 7 and 4 times greater than the corresponding values of PMA60G0. Evidently, the obtained PMAG hydrogels were significantly stronger than a single-network PMA hydrogel, but they were also comparable in terms of compressive mechanical properties to the other strong hydrogels (with a WC > 50%) reported in the literature (Table 2 and Figure 7).

**Figure 8:**
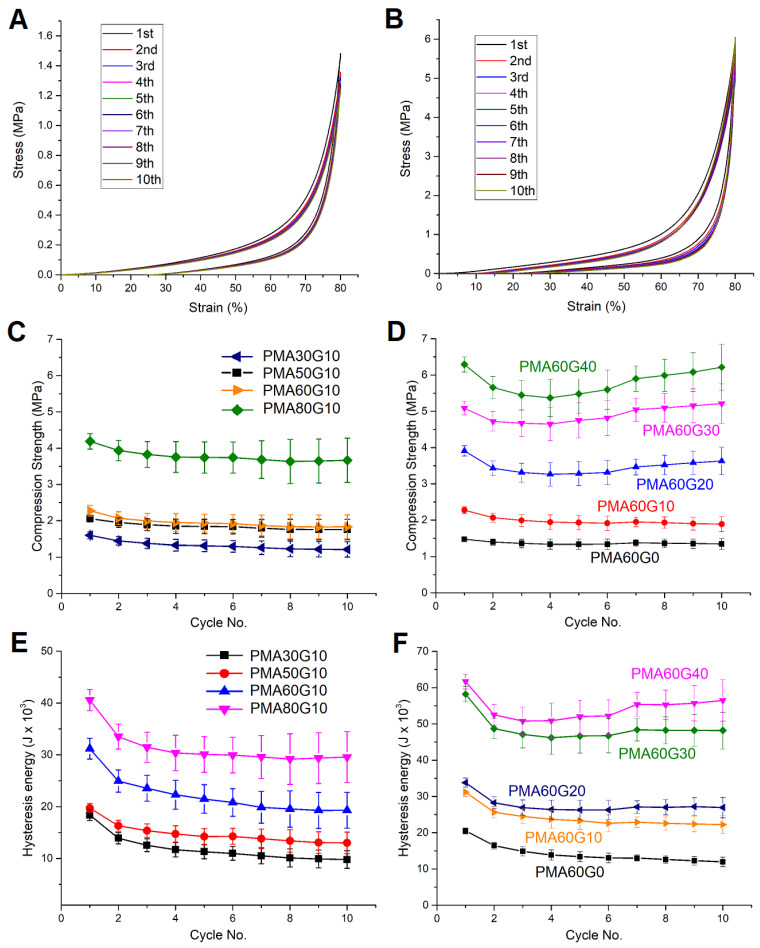
Typical compression stress–relaxation curves for 10 consecutive cycles of PMA60G0 (**A**) and PMA60G40 (**B**). Compression stress at 80% strain in 10 cycles for hydrogels with different MA (**C**) and gelatin (**D**) contents. Hysteresis energy after 80% strain in 10 cycles for hydrogels with different MA (**E**) and gelatin (**F**) contents.

To evaluate the fatigue resistance and shape recovery properties, the hydrogels were further subjected to a cyclic uniaxial compression test. Typical stress–strain curves for 10 consecutive cycles and the trend in maximum stress and energy dissipation in each cycle are summarized in Figure 8. The hysteresis loops and loss factors decreased during the second compressive cycle for all hydrogels, which indicated that the internal network of the hydrogels was partially disrupted. During the subsequent compression cycles, the values of *σ_c_* and hysteresis energy (*H*) either reached a plateau or recovered to a certain extent, depending on the composition of the hydrogels. The increase in MA content led to an increase in fatigue resistance. For example, the values of *σ_c_* and *H* of PMA30G10 were reduced to 79.0% and 55.5%, respectively, compared to the initial cycle. On the other hand, the corresponding values of PMA80G10 fell to 91.4% and 76.1% compared to the initial cycle. The increase in MA content (and thereby increased initiator content) led to the creation of a higher number of macromolecular chains, which led to an increased density of sacrificial bonds and tougher hydrogels. Nevertheless, the hydrogels from the PMAxG10 series did not exhibit significant recoverability during the 10 cycles of the test. On the contrary, the PMA60Gy hydrogels (y = 20, 30, and 40) exhibited significant recoverability of *σ_c_* and moderate recoverability of *H*. Compared to the pure-PMA hydrogel (PMA60G0), which had values of *σ_c_* and *H* that were reduced to 83.9% and 59.0% after the 10th cycle, respectively, the *σ_c_* of PMA60G40 completely recovered after the 10th cycle despite falling to 87.7% during the 4th cycle, while *H* decreased to 83.8% during the 3rd cycle and recovered 92.7% of the initial value after the 10th cycle. These results clearly suggest that the increase in gelatin content had a stronger influence on the fatigue resistance and recoverability in comparison to the increasing MA content.

**Figure 9:**
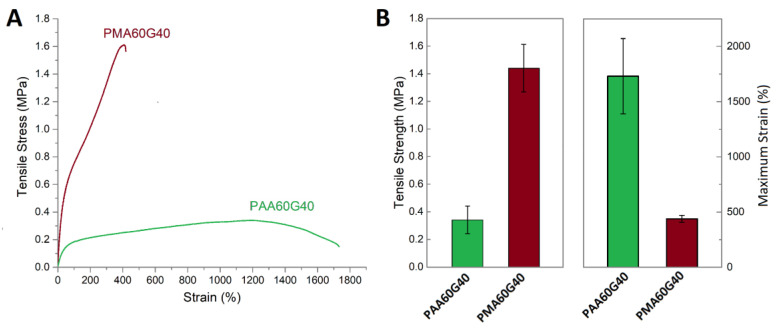
Typical stress–strain curves for PMA60G40 and PAA60G40 hydrogels (**A**). Comparison of the tensile strength and strain for PMA60G40 and PAA60G40 hydrogels (**B**).

Evidently, the introduction of the gelatin drastically improved the mechanical characteristics of the PMA hydrogels, which, in the case of the synthesized PMAG hydrogels, were comparable with tough hydrogels reported by the other researchers (Table 2 and Figure 7). The pure-PMA hydrogel was physically crosslinked through physical bonds and could form a three-dimensional network; however, the incorporation of gelatin significantly increased the number of dynamic and permanent crosslinks, which increased the mechanical properties. Interestingly, the XRD results indicated an absence of gelatin triple-helix junctions, which means that the gelatin did not form an independent crosslinked network but, rather, was incorporated into the PMA network. However, the increased mechanical and dimensional recoverability, as well as decreasing EWC, indicated an increase in crosslinks in the structure of the PMAG hydrogels. The FTIR results and the opaqueness of the hydrogels suggested the formation of uniformly dispersed phase separation and polymer-rich domains with increased hydrophobic interactions, which presumably acted as permanent crosslinking points in the structure. Zhang et al. investigated gelatin/poly(MA-co-AA) hydrogels and assumed that the increasing MA content forced the folding of chains and the formation of chain-dense regions with increased hydrophobic interactions and stabilized hydrogen bonds [11]. On the other hand, when the MA/AA ratio was low, the effect was less pronounced as fewer hydrophobic domains could be formed. A similar structure could be proposed for the hydrogels obtained in this work, where the increase in gelatin content led to a higher degree of hydrophobic interactions and crosslinking, which eventually led to better mechanical properties and faster chain relaxation and recoverability. In addition, the increase in gelatin content also increased the number of hydrogen bonds formed between gelatin and PMA molecules, contributing to a higher density of sacrificial bonds and significantly higher energy dissipation and toughness. To highlight the effect of hydrophobic bonds on the physical and mechanical properties of hydrogels, a reference PAA60G40 hydrogel was prepared by replacing the MA monomer with acrylic acid (Figure 9). The PAA60G40 hydrogels exhibited a tensile strength that was an order of magnitude lower while being significantly more deformable, which confirmed the important role of hydrophobic interactions in the crosslinking and improvement in mechanical properties.

### 2.7. Self-Healing

**Figure 10:**
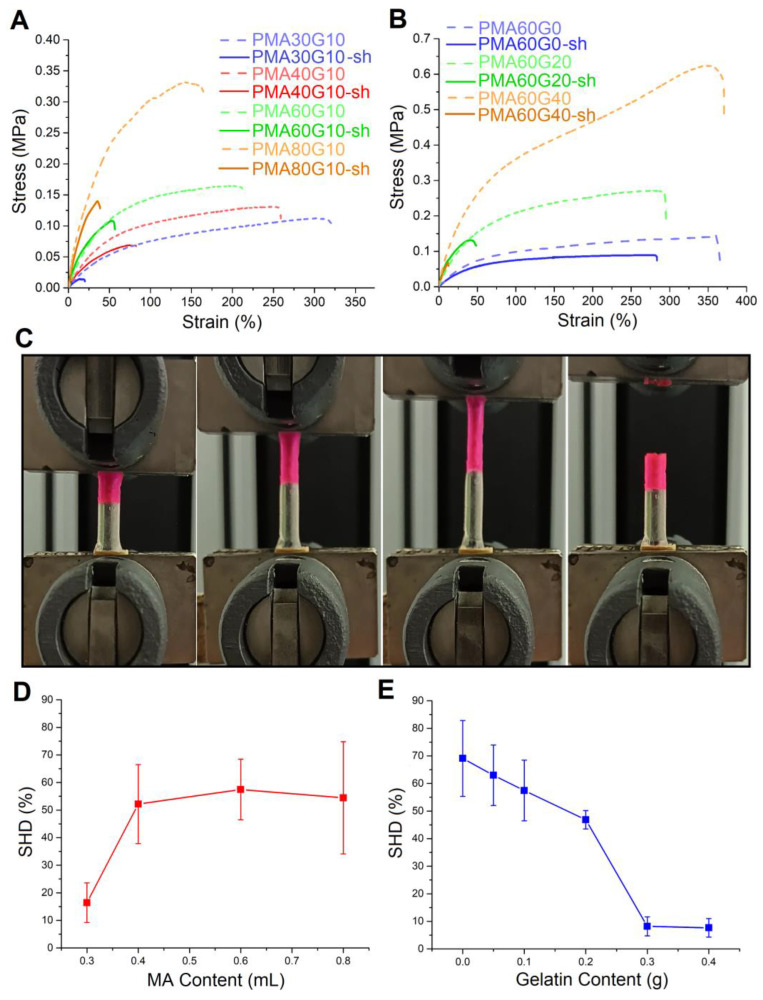
The comparative stress–strain curves of raw and self-healed (sh) samples for hydrogels with fixed gelatin (**A**) and MA (**B**) contents. Photographs from tensile test of a healed PMA60G0 sample (**C**). The influence of MA (**D**) and gelatin (**E**) content on the self-healing ability of the hydrogels.

Figure 10A,B illustrate a comparative analysis of stress–strain curves between raw samples and self-healed (sh) hydrogels. In Figure 10C, images from the tensile testing of a healed cylindrical-shaped PMA60G0 sample are presented, demonstrating its excellent self-healing capability. Notably, the sample did not fracture at the healing site but instead at the clamping point, emphasizing the hydrogel’s effectiveness in self-repair. Figure 10D,E demonstrate the self-healing ability of the hydrogels. It was observed that the increase in MA content (with the gelatin content fixed) enhanced the self-healing ability of the hydrogels, while the increase in gelatin content (with the MA content fixed) had the opposite effect. For example, the values of SHD for the PMA30G10 hydrogel and PMA80G10 were 16.4% and 54.4%, respectively. The SHD values for PMA60G0 and PMA60G40 were 69.1% and 7.6%. Evidently, the gelatin/MA ratio had the greatest influence on the self-healing ability of the hydrogels. At lower ratios, the degree of crosslinking was reduced due to the lower degree of formation of hydrophobic domains. This led to an increase in the fraction of dynamic hydrogen bonds, i.e., hydrogen bonds that were not fully saturated. Consequently, molecules exhibited greater interaction with the surface of the fused hydrogel section. On the other hand, the increase in gelatin/MA ratio increased the number of permanent crosslinks (hydrophobically stabilized hydrogen bonds), which reduced the number of available dynamic bonds, therefore decreasing the self-healing potential of the PMAG hydrogels.

### 2.8. Shape Memory

**Figure 11:**
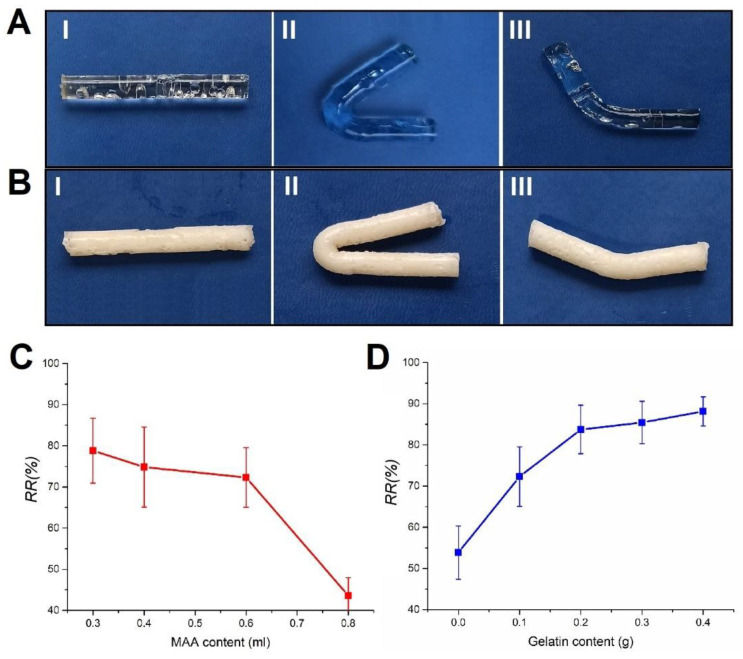
The fixation of the temporary shape and the recovery of the initial shape of PMA60G0 (**A**) (permanent shape (I), fixing to temporal shape (II), and recovery to permanent shape (III)) and PMA60G40 (**B**) hydrogels. The influence of MA content (**C**) and gelatin content (**D**) on the shape memory properties of the hydrogels.

The presence of hydrogen bonds as well as hydrophobic interactions imparted shape memory properties to the obtained PMAG hydrogels. The high-chain-density domains, promoted by hydrophobic interactions, acted as permanent crosslinking points since they were formed during the polymerization process. On the other hand, hydrogen bonds as thermally sensitive bonds acted as temporal crosslinks. The obtained structure allowed the breaking of hydrogen bonds at higher temperatures and subsequent fixation of the temporal shape at the lower temperature (Figure 11A,B).

The hydrogen bonds as switchable crosslinking points tended to dissociate and reform depending on the temperature, while hydrophobic domains exhibited stability and acted as permanent crosslinking points, which maintained the integrity of the hydrogels. The shape memory capacity of the hydrogels was assessed by calculating the recovery ratio using a bending test. As seen in Figure 11C,D, the increase in MA content reduced the recovery ratio (RR%), while the increase in gelatin content strongly promoted the shape memory properties of the hydrogels. The increase in MA content, while the gelatin content was constant, led to a higher hydrogen bonds/hydrophobic interactions ratio, diminishing the shape recovery potency of the hydrogels due to the lower number of permanent crosslinks in the structure. On the other hand, the increase in gelatin content increased the number of hydrophobic interactions and permanent crosslinks. Nevertheless, the PMA60G0 hydrogel, which did not have gelatin in the structure, also demonstrated shape memory behavior. This means that the pure-PMA hydrogels also had memory shape properties, probably due to some degree of hydrophobic interactions, as well as physically entangled chains which formed permanent crosslinks during the polymerization process.

## 3. Conclusions

Novel PMAG hydrogels with a high content of gelatin, physically crosslinked through hydrogen bonds and hydrophobic interactions, were successfully synthesized by a simple, one-pot synthesis process. The investigation of the physicochemical properties of the hydrogels indicated the formation of hydrophobic domains which acted as permanent crosslinking points and imparted great mechanical stability and shape memory properties to the hydrogels. The increase in MA and gelatin contents significantly improved the mechanical properties and thermal stability of the PMA hydrogels while retaining a relatively high level of WC. The WC of 50–90% was in the range of that of biological tissues, while the values of compression and tensile strength were significantly higher compared to a single-network PMA hydrogel. A detailed analysis and comparison of the investigated mechanical properties of similar materials was performed, and they confirmed that the obtained PMAG hydrogels were comparable to other exceptionally strong hydrogels reported in the literature. In addition, the increase in gelatin-to-PMA ratio imparted excellent shape memory properties to the hydrogels. Taken together, the obtained hydrogels demonstrated great versatility in terms of physicochemical and mechanical properties, as well as shape memory, which makes them prominent candidates for further investigations for various biomedical and industrial applications.

## 4. Materials and Methods

### 4.1. Materials

Methacrylic acid (MA) (99.5%) and acrylic acid (AA) (99%) were supplied from Merck KGaA. Type B bovine skin gelatin (gel strength 70) was obtained from Sigma Aldrich. The initiator, 2,2′-Azobis[2-(2-imidazolin-2-yl)propane] dihydrochloride (VA-044) (99.8%), was supplied by Wako Pure Chemical Industries. All chemicals were used as received.

### 4.2. Synthesis of IPN Hydrogels

PMAG hydrogels were synthesized via one-pot free-radical polymerization. Firstly, appropriate amounts of MA and gelatin were added into the reaction glass containing 1 mL of distilled water, after which the temperature of the mixture was set to 70 °C (Table 3). After 20 min of vigorous stirring, the initiator (VA-044) was added, and the mixture was stirred for an additional 3 min before it was poured into a Teflon mold and placed in an oven at 65 °C for 6 h to complete the reaction. The concentration of MA was varied from 30 to 80% (*v*/*v*), and the concentration of gelatin was varied from 0 to 40% (*w*/*v*). The initiator/MA ratio was fixed at 0.0025 (*w*/*w*). Afterward, the samples were prepared according to the testing method requirements.

### 4.3. Hydrogel Characterization

#### 4.3.1. Degree of Monomer Conversion

The degree of monomer conversion (*DC*) into the gel phase was determined using the following equation:

(1) $$DC=\frac{m_{0}}{m_{t}}$$

where *m*_0_ is the weight of xerogel obtained by the drying of the prepared hydrogels to the constant mass, and *m_t_* is the theoretical mass of all components applied in the synthesis (MA + gelatin).

#### 4.3.2. FTIR Analysis

Fourier-transform infrared spectroscopy (FTIR) spectra of the samples were recorded in the absorbance mode using a Nicolet™ iS™10 FT-IR Spectrometer (Thermo Fisher Scientific, Waltham, MA, USA) with Smart iTR™ Attenuated Total Reflectance (ATR) sampling accessories, within the range of 400–4000 cm^−1^, at a resolution of 4 cm^−1^ and in 20 scan modes.

#### 4.3.3. X-ray Diffraction (XRD)

All the samples were characterized at room temperature by XRD using an Ultima IV Rigaku diffractometer, equipped with CuKα_1,2_ radiation, using a generator voltage (40.0 kV) and a generator current (40.0 mA). The range of 5–100° 2θ was used for all samples in continuous scan mode with a scanning step size of 0.02° and at a scan rate of 10°/min, using a D/TeX Ultra high-speed detector. A Si-monocrystalline sample carrier for sample preparation was used.

#### 4.3.4. DSC

Thermal analysis of the PMA60G0 (pure-PMA hydrogel), PMA60G10, PMA60G20, PMA60G30, PMA60G40, and gelatin was performed using a differential scanning calorimeter—DSC (DSC-60 Plus differential scanning calorimeter, Shimadzu, Kyoto, Japan). Before the DSC analysis, the hydrogels were dried and ground into powder. The weight of the samples was limited to 4 ± 0.2 mg, and all DSC measurements were carried out using hermetic aluminum pans. The thermal analysis of the samples was conducted from 25 °C to 300 °C at 10 °C/min under a nitrogen purge gas flow of 30 mL·min^−1^.

#### 4.3.5. Determination of Equilibrium Water Content

The equilibrium water content (*WC*) was determined in simulated body fluid (SBF, prepared as in [52]) at 37 °C, after 24 h, using the following equation:

(2) $$WC\;\left(\%\right)=\frac{m_{eq}-m_{0}}{m_{eq}}\times 100$$

where *m_eq_* is the weight of the swollen hydrogel sample at equilibrium, and *m*_0_ is the weight of the xerogel.

#### 4.3.6. Morphological Investigations

A Tescan MIRA 3 XMU field emission scanning electron microscope (SEM), Brno, Czech Republic, operating at 20 kV was used to characterize the morphology of the hydrogels. Prior to examination, all samples were swollen to equilibrium in SBF, lyophilized, and sputter-coated with gold using a POLARON SC502 sputter coater in order to avoid electrostatic charge. In order to prepare the samples for lyophilization, the samples were frozen at −80 °C for 1 h before the procedure. The solidified scaffolds were then transferred into a freeze dryer (Beta 2–8 LD plus, Martin Christ GmbH, Osterode am Harz, Germany) and dried at −60 °C (at a pressure of 0.011 mbar) for 24 h and at −75 °C (at pressure of 0.0012 mbar) for an additional hour to remove the residual water.

#### 4.3.7. Mechanical Properties

The mechanical properties of the hydrogels were evaluated using a Universal Testing Machine AG-Xplus (Shimadzu, Kyoto, Japan), equipped with a 1000 N force load cell (force range from 0.01 to 1000 N). Hydrogels were tested immediately after the synthesis. A tensile test was performed until the failure of the sample, at a deformation speed of 20 mm/min. Specimens were half-cylindrical with a radius of 2 mm. The values of tensile strength were determined as stress values at failure, while the toughness was calculated as the area under the stress–strain curve.

Unconfined compression testing was performed on 8 × 5 mm cylindrical specimens, up to 100% deformation, at a compression speed of 6 mm/min. Automatic detection of the contact between the plate and hydrogel was performed by setting a contact force of 0.02 N. The values of compressive strength were determined as stress values at strain = 90%. The compressive modulus was calculated as the slope of the stress–strain curve from the linear region between 0% and 10% strain. At least three specimens were tested for each hydrogel, and the mean values and standard deviations were calculated. A cyclic compressive test was performed for 10 cycles up to a deformation of 80%, at a compression speed of 10 mm/min, without any waiting time between cycles. The hysteresis energy was determined from the area between the loading–unloading curves.

#### 4.3.8. Self-Healing

The self-healing behavior of the hydrogels was evaluated by tensile testing. A cylindrical hydrogel sample with a diameter of 5 mm was cut in half and subsequently healed by adhering the two cut halves, without external intervention at 25 °C for 24 h. The self-healing degree (*SHD*) of the hydrogels was calculated using the following equation:

(3) $$SHD\;\left(\%\right)=\frac{\sigma_{h}}{\sigma }\times 100$$

where *σ_h_* is the tensile strength of the healed sample, and *σ* is the tensile strength of the as-synthesized sample.

#### 4.3.9. Shape Memory Properties

The synthesized cylindrical hydrogel sample was immersed in a hot water bath at 70 °C for 2 min. The softened elastic gel was subsequently bent into a temporary U shape and fixed by immersing it in a cold water bath of 5 °C. After fixing the temporary shape, the sample was then re-immersed in hot water at 70 °C to recover its permanent shape. The shape recovery ratio (*RR*) was determined using the following equation:

(4) $$RR\;\left(\%\right)=\frac{\theta_{t}-\theta_{f}\;}{\theta_{t}}\times 100$$

where *θ_t_* is the temporarily fixed angle, and *θ_f_* is the final angle.

## Figures and Tables

**Table 1:** Review of the mechanical properties of hydrogels based on gelatin or PMA reported in the literature.

Material	*σ_t_* (MPa)	*U_t_* (MJ m^−3^)	*σ_c_* (MPa)	*E_c_* (MPa)	WC (%)
gelatin/poly(acrylamide-*co*-hexadecyl methacrylate) IPN [34]	1.48	8.83	/	/	/
gelatin/PMA photopolimerized hydrogel [20]	4.93	/	/	/	45
gelatin hydrogel soaked in (NH_4_)_2_SO_4_ [35]	4.31	/	13.01	0.46	50
gelatin/PHEMA hydrogels soaked in (NH_4_)_2_SO_4_ [38]	5.99	16.57	/	/	36
gelatin/poly(N-hydroxyethyl acrylamide) [36]	2.33	/	/	/	30
gelatin/poly (MA–*co*-AA) [11]	7	/	/	/	40
gelatin/PVA double-network hydrogel soaked in Na_2_SO_4_ [39]	1.0	3.2	283	/	80
PMA/gelatin semi-IPN hydrogel [19]	/	/	16	0.16	60
PMA/Triton X-100 [37]	3.6	20	93	30	26
**PMA60G40**	1.44	4.91	24.81	0.75	51

**Table 2:** A legend for Figure 7 detailing the composition and preparation method of the referenced hydrogels.

Reference	Composition	Preparation Method
Ugrinovic 2022 [19]	PMA/gelatin semi-IPN hydrogel	Thermally induced free-radical polymerization
He 2018 [35]	Gelatin	Soaking in (NH_4_)_2_SO_4_
Means 2019 [40]	(PAMPS)/P(NIPAAm-co-AAm) IPN	Two-step UV polymerization
Xiang 2017 [41]	PAMPS/P(AAm–AAc)	Two-step free-radical polymerization coupled with ion crosslinking
Du 2014 [42]	PEDOT/PAMPS/PAAm	Three-step polymerization
Li 2018 [43]	PNAGA/CMC	Polymerization of N-acryloyl glycinamide with CNC, and crosslinking with Fe^3+^
Gong 2003 [44]	PAAMPS/PAAm	Two-step UV polymerization
Li 2019 [45]	SF/CMCS	Chemical crosslinking followed by ethanol treatment
O’Brien 2020 [46]	Polypeptide/PEG IPN	Polypeptide synthesis followed by swelling in PEG-dithiol and UV polymerization
Huang 2007 [47]	Macromolecular microsphere hydrogels based on styrene, butyl acrylate and AA	Emulsion polymerization
Dong 2013 [48]	PAMPS/PAAm/CNTs IPN	Two-step free-radical polymerization
Wang 2019 [49]	Alginate/PAAm IPN	Free-radical polymerization coupled with ion crosslinking
Wang 2017 [50]	Star PEG vinyl sulfone	Coupling star PEG-OH with an excess of divinyl sulfone
Zhao 2018 [51]	PVA	Ionically crosslinked

**Table 3:** Feed composition.

Samples	MA (mL)	AA (mL)	Gelatin (g)
PMA30G10	0.30	/	0.10
PMA40G10	0.40	/	0.10
PMA50G10	0.50	/	0.10
PMA60G10	0.60	/	0.10
PMA80G10	0.80	/	0.10
PMA60G0	0.60	/	0.00
PMA60G5	0.60	/	0.05
PMA60G20	0.60	/	0.20
PMA60G30	0.60	/	0.30
PMA60G40	0.60	/	0.40
PAA60G20	/	0.60	0.20
PAA60G40	/	0.60	0.40

## Data Availability

The data presented in this study are openly available in article.
